# National Association of Examiners

**Published:** 1888-10

**Authors:** 


### National Association of Examiners.

The Seventh Annual Meeting of the National Association of Dental Examiners was held in Louisville, Ky., August 27th, 28th, and 29th, 1888.

The meeting was called to order by the President, Dr. Geo. H. Cushing, who stated that being no longer a member of the State Board of Examiners of Illinois, he could not be considered a member of the National Association. He therefore vacated the Chair in favor of the Vice-President, Dr. T. S. Waters, of Baltimore.

Roll call showed the following State Boards represented:

Georgia, Dr. S. B. Barfield; Illinois, Drs. C. R. E. Kirk, R. N. Lawrence: Indiana, Drs. P. G. C. Hunt, S. F. Kirk; Kentucky, Dr. A. O. Rawls; Maryland, Dr. G. S. Waters; Mississippi, Dr. W. W. Westmoreland; New Jersey, Dr. Fred. A. Levy; Ohio, Drs. J. Taft, H. A. Smith; South Carolina, Dr. G. T. S. Wright; Wisconsin, Dr. B. G. Maercklein.

At the next session Arkansas, Drs. M. C. Marshall, L. G. Roberts was added to the list.

A letter from the State Board of Wisconsin was read and placed on file. The letter asked the consideration of the National Board of the following subjects:

The licensing of first course students, this seeming to be a tacit admission that a State Board is satisfied with an inferior standard, and willing to admit into practice men who have acquired but the half or even less of the little required by the colleges. State laws which provide for examination in lieu of a diploma may be considered weak in this conception, and until the diploma is made the only basis of qualification, some uniform method should be recommended by which this doubtful practice may be overcome.

The second section of the letter is in reference to provision for temporary license to practice in the interim between meetings of the State Board.

The third section is in reference to a more uniform list of questions for examination by State Boards.

On motion, a committee of three, Drs. Taft, Koch, and Barfield was appointed, and the communication referred to them.

At the next session the committee reported in substance as follows:

They *respectfully recommend* "the discontinuance of the practice of giving permits to practice dentistry to students during the time of their college work or before their graduation;

"That any applicant for examination and license to practice dentistry between the regular sessions of the respective State Boards, may be examined during such interim by one or more members of a State Board, as may be designated by the said Board, and upon such examination being satisfactory, a permit may be issued to the applicant to practice dentistry till the next meeting of the Board, and no longer. The examination and permit shall in no case exempt the candidate from an examination by the full Board at its next regular meeting.

They do not believe it advisable to have a uniform list of questions for examination throughout the country. They *recommend* that the State Boards embrace in their examinations the following branches, and that there be not less than ten questions on each of these branches, *viz.*: Physiology, Pathology, Histology, Hygiene, Materia, Medica, Therapeutics, Chemistry, Metallurgy, Operative Dentistry, Prosthetic Dentistry, Dental Jurisprudence. They *suggest* that each State Board formulate its own list of questions, and that the list be changed at least once each year, and that a standard of at least 75 per cent. of correct answers be required.

The report was received and adopted.

Dr. Koch asked for information as to the standing of the Dental Department of Howard University, and the Dental Department of the National University—both of Washington City. A committee of three, consisting of Drs. Levy, Rawls, and Wright was appointed, to whom the matter was referred, whose duty it was made to prepare a list of the Dental Colleges which the National Association of Examiners will recommend to the various State Boards as those whose diplomas may be accepted instead of an examination.

The committee reported a list of colleges which was accepted at the second session. At the last session, the report was reconsidered, the list was referred back to the committee, revised, presented to the Association, amended, and finally accepted, as follows:

Baltimore College of Dental Surgery, Baltimore, Maryland.

Boston Dental College, Boston, Massachussetts.

Chicago College of Dental Surgery, Chicago, Illinois.

Harvard University, Dental Department, Cambridge, Massachussetts.

Kansas City Dental College, Kansas City, Missouri.

Minnesota Hospital, Dental Department, Minneapolis, Minnesota.

Missouri Dental College, St. Louis, Missouri.

New York College of Dentistry, New York City.

Ohio College of Dental Surgery, Cincinnati, Ohio.

Pennsylvania College of Dental Surgery, Philadelphia, Pennsylvania.

Philadelphia Dental College, Philadelphia, Pennsylvania.

St. Paul Medical College, Dental Department, St. Paul, Minnesota.

University of California, Dental Department, San Francisco, California.

University of Iowa, Dental Department, Iowa City, Iowa.

University of Michigan, Dental Department, Ann Arbor, Michigan.

University of Pennsylvania, Dental Department, Philadelphia, Pennsylvania.

Vanderbilt University, Dental Department, Nashville, Tenn.

Northwestern College of Dental Surgery, Chicago, Illinois.

Louisville College of Dentistry, Louisville, Kentucky.

Indiana Dental College, Indianapolis, Indiana.

Dental Department of Northwestern University, Chicago, Ill.

Dental Department of Southern Medical College, Atlanta, Georgia.

Dental Department of University of Tennessee, Nashville, Tennessee.

School of Dentistry of Meharry, Medical Department of Central Tennessee College, Nashville, Tennessee.

ELECTION OF OFFICERS

President, Dr. T. S. Waters, Baltimore, Maryland.

Vice-President, Dr. S. T. Kirk, Kokomo, Indiana.

Secretary and Treasurer, Dr. Fred. A. Levy, Orange, New Jersey.

Adjourned to meet on the day and at the place of meeting of the American Dental Association, at 9:30 a. m., *viz.:* Saratoga, Tuesday, August 5th, 1889.

"MRS M. W. J."

